# Social Network Decay as Potential Recovery from Homelessness: A Mixed Methods Study in Housing First Programming

**DOI:** 10.3390/socsci6030096

**Published:** 2017-08-23

**Authors:** Elizabeth Golembiewski, Dennis P. Watson, Lisa Robison, John W. Coberg

**Affiliations:** 1 Department of Health Policy & Management, Fairbanks School of Public Health, Indiana University, 1050 Wishard Blvd., Indianapolis, IN 46202, USA; 2 Department of Social & Behavioral Sciences, Fairbanks School of Public Health, Indiana University, 1050 Wishard Blvd., Indianapolis, IN 46202, USA

**Keywords:** homelessness, Housing First, social networks, egocentric networks, social integration, serious mental illness, substance use disorder, mixed methods

## Abstract

The positive relationship between social support and mental health has been well documented, but individuals experiencing chronic homelessness face serious disruptions to their social networks. Housing First (HF) programming has been shown to improve health and stability of formerly chronically homeless individuals. However, researchers are only just starting to understand the impact HF has on residents’ individual social integration. The purpose of the current study was to describe and understand changes in social networks of residents living in a HF program. Researchers employed a longitudinal, convergent parallel mixed method design, collecting quantitative social network data through structured interviews (n = 13) and qualitative data through semi-structured interviews (n = 20). Quantitative results demonstrated a reduction in network size over the course of one year. However, increases in both network density and frequency of contact with network members increased. Qualitative interviews demonstrated a strengthening in the quality of relationships with family and housing providers and a shedding of burdensome and abusive relationships. These results suggest network decay is a possible indicator of participants’ recovery process as they discontinued negative relationships and strengthened positive ones.

## 1. Introduction

Nearly 70,000 individuals in the United States were recorded as experiencing chronic homelessness in 2016 (Henry et al. 2016), meaning they have (a) been continuously without housing for more than one year or experienced homelessness repeatedly within the past three years and (b) have a documented disability (including serious mental illness or substance use disorder) (U.S. Department of Housing and Urban Development 2013). This most recent census represents an approximate 35% decrease in chronic homelessness since 2007 (Henry et al. 2016). This drop has been attributed largely to the spread of the evidence-based permanent supportive housing model known as Housing First (HF), which was developed specifically to serve chronically homeless individuals with dually-diagnosed serious mental illness and substance use disorder (Tsemberis 2010). In contrast to traditional “treatment first” programming, HF utilizes a harm reduction approach that does not require sobriety of residents and emphasizes consumer choice relating to service participation (Tsemberis 2010; Tsemberis and Asmussen 1999; Watson et al. 2017). The HF approach has been shown to positively impact social, behavioral health, and physical health outcomes including: higher housing retention (Tsemberis and Eisenberg 2000; Goering et al. 2014); higher reported use of behavioral health services (Padgett et al. 2006); reduced substance use and abuse (Padgett et al. 2006; Padgett et al. 2010); and reduced emergency room visits (Sadowski et al. 2009). The intervention has also been touted for its ability to improve residents’ social integration; though, evidence to support this claim is limited. The current study adds to the nascent literature in this area by describing changes in the social networks of residents living in a single-site HF program that occurred over the course of one year. We provide a brief overview of research in the areas of social networks and support in the homeless population more generally and literature in this area focused on the HF model before presenting our methods and results.

### 1.1. Social Integration and Mental Health of Individuals Experiencing Homelessness

Prior research has demonstrated the degradation of important social connections to be both a precipitating factor and consequence of homelessness (Anderson et al. 1993; Daiski et al. 2012; Duchesne and Rothwell 2015; Fitzpatrick et al. 2000), as well as demonstrating those who are experiencing chronic homelessness have small social networks with diminished capacity for social support and engagement outside of that provided by social service workers (Buck and Alexander 2006; Hawkins and Abrams 2007; Nooe and Patterson 2010; Trumbetta et al. 1999). Additionally, serious mental illness, which is highly prevalent among the chronically homeless, is itself independently associated with unstable social connections (Kawachi and Berkman 2001; Kessler and McLeod 1985; Turner and Brown 2010), with low social support having a negative impact on psychological well-being and the ability of individuals to cope with existing mental health issues (Kawachi and Berkman 2001). Factors negatively impacting social support in the homeless population range from those at the individual-level, e.g., social withdrawal due to fear of mockery or victimization (Davidson et al. 2001), to society-level forces such as high rates of unemployment (Morgan et al. 2007). Substance abuse has also been linked to negative social outcomes for this group, as it often reinforces social ties to individuals who engage in drug abuse and illegal activity (Alverson et al. 2000; Drake et al. 2002).

Expanding and strengthening social networks has potential to improve mental health outcomes for individuals experiencing homelessness (Padgett and Drake 2008). Indeed, previous research has demonstrated the stress buffering effects of supportive social relationships can improve the well-being of people living with a mental illness (Kawachi and Berkman 2001; Turner and Brown 2010). Furthermore, the recovery paradigm guiding current mental health and substance use disorder services highlights the importance of positive and nurturing social ties for behavioral health rehabilitation (Alverson et al. 2000; Anthony 1993; Laudet and Humphreys 2013; Laudet and White 2008). As such, investigation of interventions with potential to improve social integration, such has HF, can improve our understanding of the recovery process.

### 1.2. Social Integration among Housing First Residents

An even smaller body of literature has examined the social connectedness of formerly chronically homeless individuals in the context of HF programming. Findings from this research, though mixed, lean in a positive direction regarding the intervention’s ability to facilitate social integration and support. Yanos has published three studies with various colleagues that have investigated different aspects of community integration (i.e., the degree to which an individual is physically, psychologically, and socially embedded in their community) among HF residents (Yanos et al. 2004, 2007, 2012). While these studies overall demonstrate improved social connections for most HF programming recipients, they also highlight various factors that can attenuate the establishment of meaningful relationships. For instance, single-site (i.e., congregate) housing that encourages interactions with fellow program participants over community activities (Yanos et al. 2007), not feeling welcome in one’s neighborhood (Yanos et al. 2004), and having only lived in a neighborhood for a short duration of time (Yanos et al. 2012) were all noted barriers that seemed to impact community integration independently of mental health and substance abuse. More recent findings from the At Home/Chez Soi study (a randomized control trial of HF across five Canadian cities) further support the notion that the structure of HF services may impact social integration, as the researchers found participants living in multiple-site (i.e., independent) apartments experienced higher levels of social isolation but were more physically and psychologically integrated into the larger community than those in single-site programs (Patterson et al. 2014; Stergiopoulos et al. 2014).

Additional research has provided some evidence for HF programming’s positive impact on residents’ social networks and support. Padgett et al. (2008) found that establishing new social ties was less important for residents’ mental health recovery and housing stability than avoiding previously existing negative relationships (often related to criminal activity or drug use). Specifically related to the structure of HF programming, additional work has demonstrated how the harm reduction service approach can lead to more trusting relationships between residents and staff (Watson 2012; Watson et al. 2013; Tsemberis 2010). Using quantitative and qualitative social network methods to compare HF residents with those living in treatment first programs, Henwood et al. (2015) demonstrated HF residents had a greater proportion of staff members in their networks and were less likely to maintain conflicting relationships. HF residents in their study also expressed that housing provided the stability needed to begin rebuilding broken relationships with family and friends and that they were guarded with close relationships for fear of being exploited for their new apartments. Finally, a mixed method pilot study conducted by Henwood et al. (2017), which is the most methodologically rigorous social network study of HF programming to date of which we are aware, found residents’ social networks decreased between baseline and 3-month follow-up. This change in network size was accompanied by a change in network composition, as the proportion of family members in networks increased, while the proportion of service providers decreased. Similar to Padgett et al. (2008), the analysis also demonstrated a distancing of network members who were considered negative influences in residents’ lives.

The current paper discusses results from one component of a year-long study of a new 38-unit, single-site HF program located in Indianapolis, Indiana. The program is the first in its area to follow a true HF approach, and stakeholders commissioned this study in order to understand the benefits of the model over other program types in the area. The stakeholders were particularly interested in understanding HF’s ability to improve social integration of residents, and therefore, this was a major goal of the study. The primary questions guiding this arm of the study were: (1) What changes in residents’ social network size and quality occurred over the course of the first year of the program’s services?; (2) How did residents perceive changes to their social networks and social support?; and (3) How were changes in social networks and support related to housing attainment?

## 2. Materials and Methods

Our study covered the first year of operation of the HF program introduced above. The study itself followed a convergent parallel mixed method design, meaning quantitative and qualitative data were collected and analyzed separately before comparing findings to locate areas where they overlapped (i.e., triangulation) and where qualitative findings both complemented and extended quantitative results (Creswell and Clark 2011). We collected data at baseline (i.e., within two weeks of move-in), 6 months, and 12 months, with structured quantitative interviews occurring at all three waves, and semi-structured qualitative interviews occurring in the second and third waves. The study was originally unfunded, and six-month data collection activities and the qualitative component were added after baseline data collection because funding was made available at that point in time.

### 2.1. Setting and Participants

The program itself was a collaboration between a local community mental health center, a homeless day shelter operatin its own street outreach team, the city housing authority, and a private developer. Program staffing included a property manager, a maintenance worker, two case managers, and a member of the street outreach team. The purpose of including the street outreach worker was to ensure residents would have a familiar face they could turn to for assistance during their first year of transitioning to domiciled living. Staff were generally scheduled to work between the hours of 8am and 8:30pm on weekdays and 1:30pm to 10pm on weekends. Fidelity data were collected at four separate time points as part of technical assistance being provided to the agency through a parallel study (Watson et al. 2014). These data indicated the program’s structure and operations were consistent with strong HF practice (e.g., low-barrier program access, harm reduction guided services, and reduced service requirements, among other important program elements).

Consistent with the overarching HF philosophy that does not require service participation, the program’s residents were not required to participate in any arm of our study. Program staff informed all residents about the study during their intake and provided the names and contact information of interested individuals to the researchers. Researchers than followed up with interested residents to provide further explanation before requesting participation and obtaining consent. In the second and third waves, we contacted individuals who participated in previous interviews using information they had provided at baseline. Due to the instability of resident mobile phone service, we placed fliers under doors to inform them of follow-up interviews. We also placed fliers under the doors of residents who did not participate in previous interviews in order to request their participation in subsequent ones.

### 2.2. Procedures

Structured interviews were computer-assisted (Gravlee 2002) and employed a web-based electronic data capture tool (Harris et al. 2009). These interviews lasted between 30–60 minutes depending on the participant’s individual engagement and capacity. Qualitative interviews were conducted immediately after 6- and 12-month structured interviews and they were audiotaped and lasted between 20–40 min. Trained researchers completed all interviews on-site in a private area. We provided residents with a $25 grocery store gift card for each interview (qualitative and quantitative) and entered them into a drawing for a $100 grocery store gift card at each time point. Our university’s institutional review board approved all procedures.

### 2.3. Quantitative Measures and Analysis

Topics covered in the structured interview included housing history, employment and finances, physical and mental health, sexual history and behavior, prior history of trauma and criminal behavior, and opinions regarding the program. Our primary social network measure was the *Important and Health Matters Social Network Battery* (Pescosolido and Wright 2004; Wright and Pescosolido 2002; PhenX Toolkit 2015). This instrument is a name generator designed to collect information on participants’ egocentric networks, which refers to alters (i.e., people) in the network of an ego (i.e., focal person of the interview). The instrument generates names of alters through eight questions asking with whom the participant discusses important and/or health matters in their lives. Participants in this study were allowed to mention up to six alters per question and a single alter could be mentioned multiple times. In the first interview, we asked participants to tell us only about relationships that existed prior to move-in so that we could obtain a true baseline. We also collected additional information on each alter. We asked for each alter’s gender and whether they were the same or a different race from the ego. We assessed the ego’s type of relationship with the alter by asking “How are you connected to this individual?” and offered a variety of relationship types they could choose from (e.g., family, friend, service provider, etc.). To assess closeness of the relationship, we asked “How close are you to this person?”, with the available response choices of “Not very close,” “Sort of close,” and “Very close.” To assess frequency of contact, we asked “How often do you see this person or talk to them over the phone or video chat?” with the response options of “Rarely,” “Occasionally,” “Frequently,” and “Very frequently.” For all multiple response questions, participants could also answer “I don’t know”.

We computed descriptive statistics for the ego, alter, ego-alter tie, and network levels of analysis. Alter/tie measures are included for both baseline and 12-month follow-up, while ego descriptive characteristics refer to baseline measurement only. Network variables of interest included average network size, density (a measure of connectedness among alters in the network), effective size (number of ties the ego has to alters who are not connected to other alters), efficiency (a function of effective size adjusted for overall size of the network and describes the proportion of an ego’s ties that are non-redundant), proportion of female alters, proportion of alters who were the same race as the ego, mean closeness, and frequency of contact (Halgin and Borgatti 2012). Our primary analysis focused on changes in network measures between baseline and 12-month interviews. Due to the small sample size, we used non-parametric Wilcoxon signed-rank procedures to test for significant differences between time points. We calculated all network measures using E-NET Version 0.41 (Borgatti 2006), and all subsequent statistical tests were carried out in SPSS 24.0 (IBM Corp., Armonk, NY, USA).

### 2.4. Qualitative Interview Focus and Analysis

Major topics covered in qualitative interviews included health, social relationships and support, and program satisfaction and perceptions. When inquiring about social integration and support, we primarily sought to understand how participants’ social networks had changed since moving into their apartments and the reasons behind those changes.

We employed a combined deductive-inductive approach to qualitative data analysis where we first identified areas of the transcripts corresponding to the primary research questions (the deductive component) and then developed a list of grounded codes based on thematic analysis of the data pertaining to each question (the inductive component; Thomas 2006; Charmaz 2014). We then applied the codes to corresponding sections using a content analysis approach (Kuckartz 2014). We established agreement between two coders (DPW and LR). To do this, 25% of transcripts were selected and coded separately by the two researchers. After independent coding, the researchers met to compare their results and discussed and revised the codes until consensus was reached. After consensus, the researchers divided and coded the rest of the transcripts separately (Charmaz 2014). The final analysis was completed by a single researcher (DPW) who conducted two levels of analysis. First, he quantified codes reflecting support type, source of support, and changes in support (for those who completed both qualitative interviews) to understand their frequency of occurrence in the dataset. He then grouped the codes into higher-level themes in order to better understand the impact of social support on participants’ lives (Charmaz 2014; Kuckartz 2014).

## 3. Results

A total of 32 residents participated in the baseline interviews: their demographic and background characteristics are displayed in Table 1. The average age of participants was approximately 48 years (with youngest being 29 and the oldest 66), and they were majority male (78%) and Black (59%). Most participants had either never been married (63%) or were divorced (31%). Related to education, most (66%) had a high school education or less, and a few (16%) had attained a post-secondary degree. The average number of times participants had experienced homelessness was 3 (with a range of 1 to 20 times), and the average length of the longest period of homelessness experienced was 6 years (with a range of 0.5 to 25 years). Records containing personal health information were not available to the research team; however, housing intake staff informed us all residents had one or more behavioral health issue. Among study participants, 29 individuals (91%) informed interviewers they had either been previously diagnosed with a psychiatric disorder and/or had answered questions indicating symptoms consistent with moderate to high substance use disorder (the three individuals who did not answer questions indicating any behavioral health issues either stated they refused to answer or that they did not know the answer to most of these questions). All individuals who completed 6-month and 12-month interviews reported a psychiatric diagnosis or answered questions indicating moderate to high symptoms of substance use disorder.

We experienced high participant attrition in subsequent data collection activities with only 18 and 16 residents completing the 6- and 12-month interviews respectively. While 2 residents had passed away during the study and 2 were evicted, the others with whom we were unable to follow-up with did not return messages or were away from the apartment during researchers’ regular visits to the building (weather was consistently mild during the year, and staff informed us that residents often enjoyed being outdoors). We conducted an ad hoc analysis of the data to determine if there were any significant demographic or behavioral health differences between those who only completed the baseline interview and those who participated in subsequent interviews, and we found no significant differences.

### 3.1. Quantitative Results

Because of participant attrition, our quantitative analysis only utilizes baseline and 12-month data for the 13 individuals who completed both interviews. Table 2 presents descriptive network characteristics at both time points. As shown, networks shrunk in size (from 3.38 alters at baseline to 2.38 alters at 12 months) while density increased. On average, networks at 12 months were comprised of a higher proportion of females and those of the same race as the participant than they were at baseline. There was no observed change in closeness, but the average frequency of contact increased significantly (*p* < 0.05) with a moderate effect size (r = 0.38).

**Figure 1:**
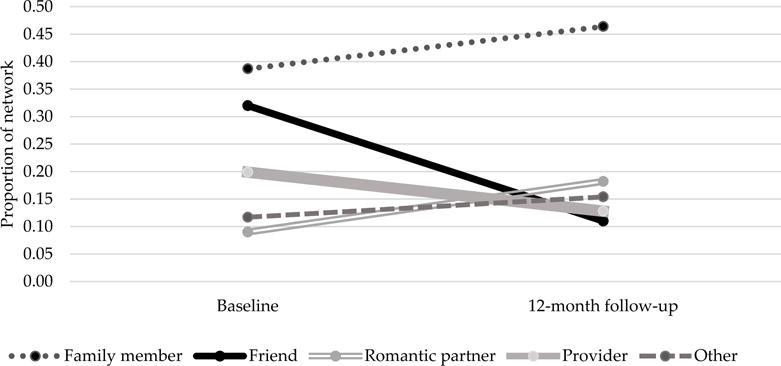
Network composition over time by proportion of relationship type (n = 13). * “Other” includes alters identified as neighbors, coworkers, clergy, fellow church members.

Figure 1 displays changes in network composition by proportion of specific relationship types embodied by alters (all results reflecting network change refer to individuals, rather than roles represented. in the network). The proportion of network alters who were family members increased from 0.38 at baseline to 0.46 at 12 months and was higher across both time points than any other single alter category. The proportion of networks composed of romantic partners also increased, doubling from 0.09 at baseline to 0.18 at 12 months. The average proportion of network members who were friends and providers decreased from baseline to follow-up (0.32 to 0.11 and 0.20 to 0.13 respectively).

**Figure 2:**
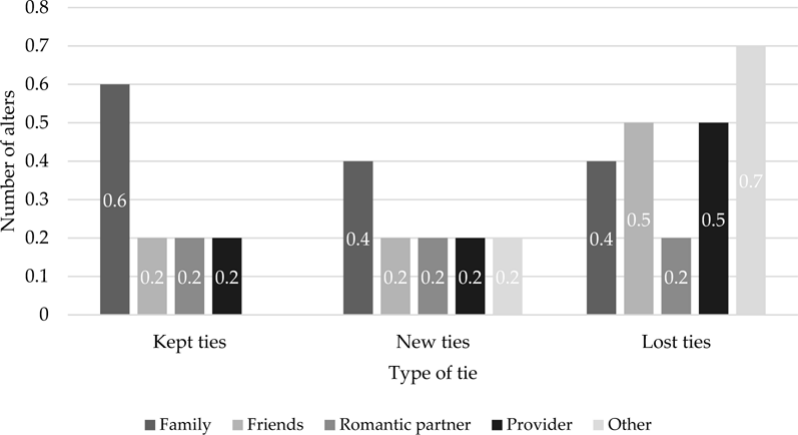
Tie churn by alter type from baseline to 12-month follow-up (n = 13). * “Other” includes alters identified as neighbors, coworkers, clergy, fellow church members.

Figure 2 supplies information on types of network alters who were retained, lost, or gained from baseline to follow-up as a measure of tie churn. Family members were most likely to be retained as kept ties from baseline to follow-up, while the most common types of lost ties at follow-up were with friends, providers, and “other” alters.

### 3.2. Qualitative Findings

Findings from qualitative interviews provided a more nuanced understanding of changes that occurred in participants’ social networks. A total of 20 participants participated in these interviews, with 12 participating in both 6-month and 12-month interviews. All qualitative interview participants also participated in structured quantitative interviews.

#### 3.2.1. Types and Sources of Participants’ Social Support

We categorized each type of social support participants mentioned as being either: (a) emotional and interactional support (i.e., a relationship an individual receives some form of empathy, compassion, or genuine caring from and/or someone the person spends time with); (b) instrumental support (i.e., tangible aid or services); or (c) negative support (i.e., burdensome and/or abusive relationships). Table 3 summarizes how many participants discussed each of these categories of support and the number of participants who mentioned specific sources of support. Instrumental support and emotional and interactional support were mentioned by about the same number of participants (12 and 10 respectively), while negative support was only mentioned by 8 individuals. Relationships with friends, neighbors, and professionals/providers were mentioned by all 20 participants, while only 15 mentioned family, 12 mentioned romantic relationships, and 2 mentioned relationships with acquaintances from church (they did not discuss these individuals as friends).

**Figure 3:**
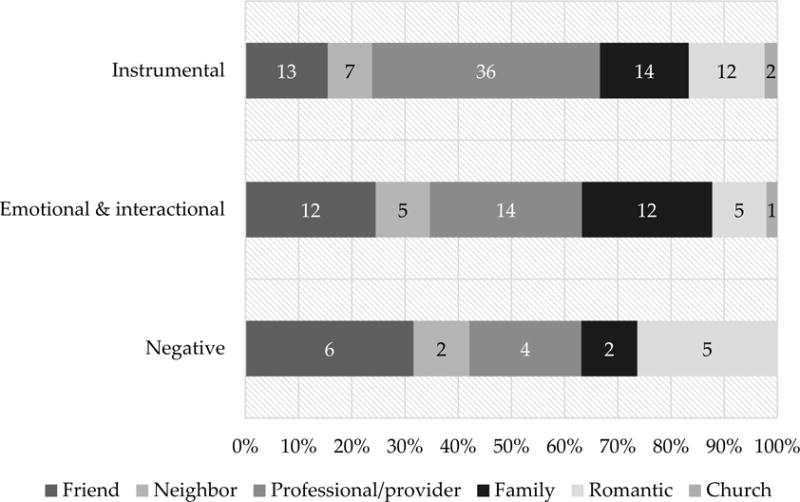
Proportion of mentions of each source of support by support type (n = 20).

Figure 3 shows the proportion of times each relationship source was mentioned by the type of social support they were discussed as providing. Instrumental support was primarily discussed in the context of professional/provider relationships, with most of these discussions centering on HF staff. As one participant described:

She [HF staff member] makes sure appointments are scheduled. She makes sure that I make my appointments. I got bus passes if I need it … She’s hooked me up with different groups and things going on, different pantries when I didn’t have my food stamps … She’s a life saver! (Male, age 50, 6-month interview)

As demonstrated by the above quote, participants often described how professionals/providers assisted them in various ways such as accessing services, dealing with legal issues, obtaining hygiene items, filling out paperwork, applying for benefits, and helping run small errands.

Friends, professionals/providers, and family were almost equally represented in discussions of emotional and interactional support, with friends primarily discussed as people to spend time with and professionals/providers primarily discussed as providing emotional support by listening to their problems or showing concern. Speaking about staff, one participant stated:

When my back’s out, I just stay in the apartment. They [HF staff] express their concern, and then, when they do see me they [say], “Man, I hope you’re alright. I see you’re feeling better, you’re up and about,” and stuff like that. There’s the concern, and it’s a truthful kind of thing. It’s not like they’re just doing it because it’s their job; they really care. (Male, age 48, 12-month interview)

Family members were different from friends and providers, as they were discussed in terms of both spending time and providing emotional support:

Because they [family] come over, we laugh and kick it and, you know, they go on about their way. It’s good. It’s pretty good. Feels good. They’re real happy for me, real happy for me. (Female, age 48, 12-month interview)

Friends and and romantic partners were the most frequently discussed sources of negative support. Examples of negative support from friends included people who a participant did not want to be around because of excessive drug and/or alcohol use, and people who took advantage of the relationship in some way such as stealing, constantly asking for money using them for their apartments. Similar issues were discussed when speaking about negative support in romantic relationships; however, in one instance a participant discussed a relationship that was marked by jealously:

I really didn’t have no friends because I had an abusive girlfriend, and I wasn’t allowed [by her] to have friends. She thought I was having sex with everybody I came in contact with.” (Female, Age 29, 6-month interview)

#### 3.2.2. Changes in Participants’ Relationships

Interview participants discussed a variety of changes in relationships that occurred over the course of the year. Positive and negative changes in relationships with friends, romantic partners, family, and professionals/providers were all noted.

When discussing *making new friends*, there was a sentiment that it was somewhat easier to do so since moving in the building:

New [friendships], yea. Before like I really kept people at a great distance for a long time … I probably got like two people that I feel really close to [since being housed]. (Female, age 51, 6-month interview)

Discussion of relationship changes related to friends often overlapped with those of other residents because many of the friendships participants developed and lost were with others living in the building. Highlighting this, one participant stated she felt it was easier for her to develop friendships since moving into the building because of the availability of her new neighbors:

Now [since moving] I have relationships with people … it’s hard to get to know somebody at a shelter. I mean, here [in the building], there are more people to pick from … More people I’d be likely to be friends with. (Female, age 58, 12-month interview)

Another common theme was the *development and subsequent backing away from friendships* with neighbors in the building who engaged in behaviors to be avoided or because they were viewed as different in some way that made them undesirable to be around. For instance, one participant discussed how she stopped engaging with some of her neighbors who she felt were a bad influence:

You know, I think there’s people [other residents] that are kind of a burden and things…I’ve kind of gotten out of the circle of the people that are drinking constantly and everything. I still associate with them, but I’m not like hanging out with them, getting drunk with them and stuff. [She associates] … with more positive people, and, you know, people I could trust more. You know, for a while I was letting about anybody in my apartment: they were stealing from me and stuff, and I kind of cut off people that I don’t trust anymore … (Female, age 48, 6-month interview)

Despite the problems this participant has with others in the building, she stated they still interact, though she avoids some of the situations and behaviors (i.e., “drinking constantly and everything”) she would rather not be a part of. The discontinuation of problematic friendships was not just limited to friends living in the building, as demonstrated by one participant’s exchange with an interviewer detailing how he dissolved two friendships with people outside of the building:

Interviewer: Those relationships, have they changed for the positive, or the negative?

Participant: Two of them for the negative.

Interviewer: Okay. Why is that?

Participant: Because one of them came in here and stole something from me. The other one … He thinks I’m supposed to believe everything he says …

(Male, age 63, 12-month interview)

Regarding romantic relationships, while there were a few discussions of these developing after a participant was housed (some of which formed between residents), there were more examples of *preexisting romantic relationships that ended*. In most of these cases, relationships had ended because the participant viewed their partner as unstable, abusive, or influencing them negatively in some way, as in the case of two participants who stated they ended relationships with physically abusive girlfriends:

… I would get away from my girlfriend … It’s like when she did something, I would do it. Because if I didn’t do it, I would get beat up … It’s over. She’s even banned from [the building] … I have support here, and I’m clean, so…If I was still hanging out with her, I probably wouldn’t be in this interview today. (Female, Age 29, 6-month interview)

Another participant discussed how he discontinued a troublesome relationship with his girlfriend who “didn’t take care of her medication” and “acted like she didn’t have a medical problem” (Male, age 50, 12-month interview). Finally, one participant who previously engaged in sex work “trading sex for drugs or sex for housing” (Female, age 48, 6-month interview), including developing a relationship with a “sugar daddy boyfriend,” was able to discontinue her reliance on those types of relationships as a means of survival after she moved into the building.

Discussions of *relationships with family were largely positive*, providing examples of participants reconnecting with family members and/or increasing their frequency or duration of contact with them. In the following example, a participant discusses how he and his wife, who were homeless together on the street and now live in the same apartment, reconnected with family after being housed:

Yeah, we’re [the participant and his wife] in contact more with them [family] now. I’ve had my brother here visiting. He stayed the night once, and we’re able to do that now. So, yeah, it’s gotten better … There was really no relationship before here. When we were homeless, they [family] didn’t try to help. They just separated themselves from us. It was like, “out of sight, out of mind” kinda thing. And now that we’re here, it’s changed. (Male, Age 48, 12-month interview)

One participant attributed their reconnection with family to *new levels of trust* that were able to develop after they had been housed:

Interviewer: … in terms of your life changing since last January, how have your relationships changed?

Participant: They’re better.

Interviewer: How would you say they are better?

Participant: My family trust me now … [now that] my lifestyle has changed.

Interviewer: Okay, could you describe that?

Participant: What my lifestyle used to be? Well, I was a hustler, boosting [stealing], doing drugs, selling drugs, that type of stuff.

(Male, age 53, 12-month interview)

Discussions of professional/provider relationships were largely focused on HF program staff, and demonstrated participants felt their *relationships with staff members had “gotten better”* over time:

Yeah, I get along really well with staff … [S]ome of them knew me before I came here, so they seen the change [in the participant’s behavior]. You know, being more social and more trusting … We work together well. (Male, age 48, 6-month interview)

Elsewhere in their interview, this participant discussed how he felt relationships between staff and participants in general had improved over time because “They [staff] know more about us now and how we act”. A different participant attributed the strengthening of her relationship with staff to the work they were investing in helping her reconnect with her child:

My relationships [with staff] have grown. I don’t really know how to explain it. They’re really working with me, try[ing] to get my mental health stable and keep me clean and sober so I can get visitation with my son. Not actually get custody with my son back but get visitation. (Female, Age 29, 6-month interview)

## 4. Discussion

Overall, the findings indicate study participants’ networks (a) decreased in size while (b) increasing in quality and that (c) these changes were at least partially due to the stability the housing program provided. Quantitative and qualitative data sources each offered unique information that provided a more robust understanding of the social networks and support of residents than any one source could do alone. Table 4 demonstrates what each of these data sources provided in terms of helping to answer the research questions.

Consistent with Henwood et al. (2017), HF residents who participated in this study experienced a reduction in network size. While a reduction in the number of alters in an ego’s social network may be taken as a sign of network decay, additional results demonstrate participants in this study experienced increased quality in relationships with alters who remained, as indicated by increased frequency of contact and discussions of better or more trusting relationships. In further support of this finding, a number of relationships participants had ended were with individuals who were abusing or taking advantage of them in some way, a finding similar to those of both Padgett et al. (2008) and Henwood et al. (2015, 2017). There are likely significant benefits to discontinuing these types of relationships, as previous research has noted the potentially detrimental effects of negative social interactions on mental health outcomes (Lee and Szinovacz 2016; Lincoln 2000). Additionally, strong relationships with similar individuals may be detrimental to members of marginalized groups, as they can embed individuals who are looking to improve their lives in a “web of obligations” (Mitchell and LaGory 2002, p. 215) that can be difficult to escape. These obligations are what participants who dissolved negative relationships with friends seemed to be avoiding, whether they were aware of it or not.

Consistent with previous HF research and more general homelessness research, we found social service providers to be a significant source of support for participants (Buck and Alexander 2006; Henwood et al. 2015, 2017; Trumbetta et al. 1999). Our findings also reinforce previous assertions that HF residents develop stronger and more trusting relationships with staff (Watson 2012; Watson et al. 2013; Tsemberis 2010), though we cannot attribute these changes to any specific attribute of the HF model. While it may seem contradictory that relationships with providers improved while the proportion of providers in the network decreased, the most likely explanation is that, upon moving into housing, participants were more consistently accessing support from a small number of HF staff as they began to rely less on multiple providers in the community.

The increase in proportion of family members in participants’ networks and the discussions around rebuilding family relationships are consistent with findings of Henwood et al. (2015, 2017) and the At Home/Chez Soi study (Coltman et al. 2015), as housing provided residents with a foundation from which they could begin to interact more regularly with residents and begin to reestablish trust. It is unfortunate we did not observe an increase in supportive friendships or a reconnection with old friends as observed in Henwood et al.’s (2015) study. It is possible burdensome friends who were shed by participants will be replaced with more healthy relationships given enough time, as this would be consistent with Yanos et al.’s (2012) finding demonstrating the relationship between length of housing tenure and community integration for HF residents.

Limitations of this study include the small sample size, participant attrition, and the sampling of participants from only one housing site that was new in its application of HF practice. While these limitations cannot be ignored, this study also had several strengths. For instance, the use of both qualitative and quantitative methods provided a means of addressing limitations inherent in any one data type through the data mixing process (Creswell and Clark 2011). Like Henwood et al. (2017), we used a name generator instrument, which has generally been proven to be a valid approach to collecting egocentric data. While Henwood et al. (2017) did have a larger sample size with less attrition, we followed participants over an entire year instead of 3 months. High fidelity scores obtained by the program ease any concerns that Type III error (e.g., false attribution of results to a program that was not properly implemented) might have impacted results given the newness of the program. Regarding similarity between our sample and the larger homeless population, age, gender, and ethnicity were largely reflective of the city’s homeless population, as reflected in the most recent homeless census (Sankari and Littlepage 2016). When compared to national figures, our sample had a higher ratio of African Americans (66% vs. 39%) but was generally similar in relation to other demographics (Henry et al. 2016). Finally, previous research has demonstrated differences in community integration between participants in single- and multiple-site HF programming (Patterson et al. 2014; Stergiopoulos et al. 2014; Yanos et al. 2007), and the impact of housing on social networks might have differed for our participants had they been housing in independent apartments. As such, further investigation of the potential effects of housing structure on social networks is warranted.

## 5. Conclusions

In sum, it seems as though casting off burdensome relationships and developing and strengthening supportive ones is a potential component of HF residents’ recovery process as it relates to homelessness that should be further investigated. Regarding implications for HF practice, providers should anticipate network atrophy and provide appropriate supports to assist residents in adjusting to changes in social relationships. Being aware of individual residents’ desires to avoid negative relationships could lead to better tailored service plans to support them in their decisions (e.g., developing strategies to avoid users of drugs and alcohol, preventing emotionally and physically abusive individuals from entering the building, and encouraging residents to interact with family and friends who are positive sources of support).While this study focused on the HF model, it supports results of previous research demonstrating the burden close relationships can place on members of marginalized groups. Future work should seek to verify these assumptions in other HF programs while addressing the limitations of the current study.

## Figures and Tables

**Table 1:** Participant characteristics at baseline (N = 32).

Variable	n	%	Mean	SD	Range
**Sex**					
**Male**	25	78.1			
**Female**	7	21.9			
**Age [Years]**			48.3	9.8	29.0–66.0
**Race**					
**Black**	19	59.4			
**White**	13	40.6			
**Educational level**					
**High school or less**	21	65.6			
**Some college**	6	18.8			
**Associates or technical degree**	4	12.5			
**College graduate**	1	3.1			
**Marital status**					
**Never married**	20	62.5			
**Divorced, separated, or widowed**	10	31.3			
**Married**	2	6.3			
**Number of homeless episodes**			3.1	3.9	1.0–20.0
**Longest episode of homelessness [Years]**			6.1	6.5	0.5–25.0
**Self-reported psychiatric diagnosis**	18	56.3			
**Moderate to high substance use disorder symptoms**^a^	26	81.3			

a Measured with Screening and Severity of Substance Use Problems questionnaire (Center for Substance Abuse Treatment 1994).

**Table 2:** Characteristics of participants’ networks changes between baseline and 12 months (N = 13).

Variable	Baseline			12 months			Percent Change

	Mean	SD	Range	Mean	SD	Range	
Network size	3.38	1.98	1.0–8.0	2.38	1.19	1.0–5.0	−29.6%
Network density^a^	0.65	0.29	0.1–1.0	0.79	0.29	0.0–1.0	+21.5%
Effective size	1.72	0.71	0.1–3.3	1.37	0.78	1.0–3.0	−20.3%
Efficiency	0.49	0.23	0.2–1.0	0.45	0.21	0.3–1.0	−8.2%
Proportion female	0.58	0.36	0.0–1.0	0.68	0.34	0.0–1.0	+17.2%
Proportion same race	0.93	0.21	0.3–1.0	0.98	0.58	0.8–1.0	+5.4%
Mean closeness^b^	2.74	0.32	2.0–3.0	2.74	0.34	2.0–3.0	0.0%
Mean contact^c^	2.48	0.49	1.7–3.0	2.80	0.24	2.3–3.0	+12.9%*

a Controlling for network size, the proportion of alters in an ego’s network who are connected to each other;

b Ego closeness to alter was measured as “1 = Not very close,” “2 = Sort of close,” or “3 = Very close”.

c Ego frequency of contact with alter was measured as “1 = Rarely,” “2 = Occasionally,” “3 = Frequently,” or “4 = Very frequently”.

* *p* < 0.05.

**Table 3:** Frequency of residents discussing different types and sources of support in qualitative interviews (N = 20).

Variable	n
**Support type**	
Instrumental	12
Emotional & interactional	10
Negative	8
**Support Sources**	
Friends	20
Neighbors	20
Professional/Provider	20
Family	15
Romantic	12
Church	2

**Table 4:** Comparison of quantitative results and qualitative findings.

Research Questions	Quantitative Results	Qualitative Findings	Conclusions
1. What changes in social network size and quality occurred over the course of the first year of services?	Networks decreased in size, while increasing in density and frequency of contact between ego and alters.	Loss of alters not seen as problematic or was due to shedding of negative relationships.	Decrease in network size was due largely to shedding of negative relationships.
2. How did residents perceive their social networks and social support to change?	Proportion of network alters who were family members and romantic partners increased, while providers and friends decreased. Family members were the most likely to be retained in networks, while the most likely to be lost were friends, providers, and other relationships.	While some new friendships and romantic relationships were added, participants largely discussed strengthening of relationships with family and staff and shedding of abusive relationships.	While changes in network composition led to some lost relationships, relationship quality with those who remained in the network improved.
3. How were changes in social networks and support related to housing attainment?	N/A	Participants discussed more opportunities to make friends, being able to visit with family more, family and staff developing trust in them, and discontinuing previous friendships and romantic relationships that were negative because they were able to recognize the abuse or no longer needed their support to survive.	Housing provided individuals with more opportunities to engage with family and friends, while also providing stability from which trusting relationships could grow. Housing also provided residents the stability they needed to discontinue abusive relationships.
